# An experimental study of the correlation between P-wave velocity and the physical properties of weakly cemented formations

**DOI:** 10.1038/s41598-023-48783-1

**Published:** 2023-12-11

**Authors:** Dong Lin, Yuhuan Bu, Huajie Liu, Chang Lu, Shenglai Guo, Hongzhi Xu

**Affiliations:** 1 China University of Petroleum (East China), Qingdao, People’s Republic of China; 2 CNPC Engineering Technology Research Company Limited, Tianjin, People’s Republic of China

**Keywords:** Engineering, Physical oceanography

## Abstract

Deep water and shallow layers mostly feature weakly cemented formations, with complex geological structures, geological looseness, susceptibility to collapse. In order to obtain information on weakly cemented formation materials, weakly cemented argillaceous siltstone is simulated as the research object and the focus is on analysing the influence of ultrasonic frequency, density, particle size (porosity), and compressive strength on P-wave velocity and establishing the correlation relationship between longitudinal wave velocity and each parameter through indoor simulation experiments. The results showed that there is a linear relationship between P-wave velocity and ultrasonic frequency in terms of positive correlation as well as compressive strength. The nonlinear relationship between P-wave velocity and particle size (porosity) is a negative correlation, while the nonlinear relationship between sound velocity and density is a positive correlation. In addition, the influence of core height on P-wave velocity is analysed; it is found that as the core height increases, the velocity slightly decreases, and each ultrasonic frequency has an ultimate height for sound wave penetration. Through the response relationship between ultrasound and the physical properties of weakly cemented formations, indirect acquisition can be achieved, which is of great significance for the development of oil and gas in weakly cemented formations.

## Introduction

With the shift of oil exploration and development from land to the ocean, deep water in the ocean has become an important field of oil and gas exploration. The complex geological environment of deep water and shallow layers poses numerous technical problems for oil and gas development^1–4^, such as weak cementation in deep water and shallow layers, low strength, and susceptibility to formation collapse and wellbore collapse during drilling. Therefore, it is particularly important to study the characteristics of shallow and weakly cemented formations in deep water. However, it is very difficult to directly obtain material data from weakly cemented formations in deep water and shallow layers, so further analysis of formation characteristics using ultrasonic response has become a practical and feasible method.

To clarify the response relationship between ultrasound and weakly cemented formations, it is first necessary to understand the factors that affect the ultrasound response. Many scholars have conducted studies in this area. Bin et al.^5^ argued that the P-wave velocity of weakly cemented sandstone with different particle sizes decreases with increasing particle size. Tutuncu et al.^6^ made weakly cemented sandstone using quartz as particles and studied the effect of permeability on elastic wave velocity. Song et al.^7^ studied the effects of factors such as sandstone particle size, temperature, and pressure on the wave velocity of loose sandstone and proposed a sound wave velocity model for loose sandstone. Zhao et al.^8^ used artificial rock samples to study the relationship between the wave velocity of loose sandstone and factors such as porosity, cementation degree, clay, temperature, fluid type, and fluid saturation. Zhou et al.^9^ analysed the influence of different saturation levels of water on the acoustic velocity of weakly cemented formations. Ultrasonic longitudinal and transverse wave velocities and densities were measured under variable temperature and pressure conditions by Ma et al.^10^. The porosities of the samples ranged from 2 to 27%, and the measurement data were used to obtain the pressure and temperature correction formulae for the longitudinal and transverse wave velocities.

Currently, less research has been done on weakly cemented formations, and comprehensive research has not yet been conducted in this area. On the basis of previous research, this article examines the relationship between ultrasonic longitudinal wave velocity and frequency, weakly cemented formation characteristics, and testing rock sample response, which is of great significance for obtaining material data from shallow weakly cemented formations in deep water.

## Experimental setup

**Figure 1:**
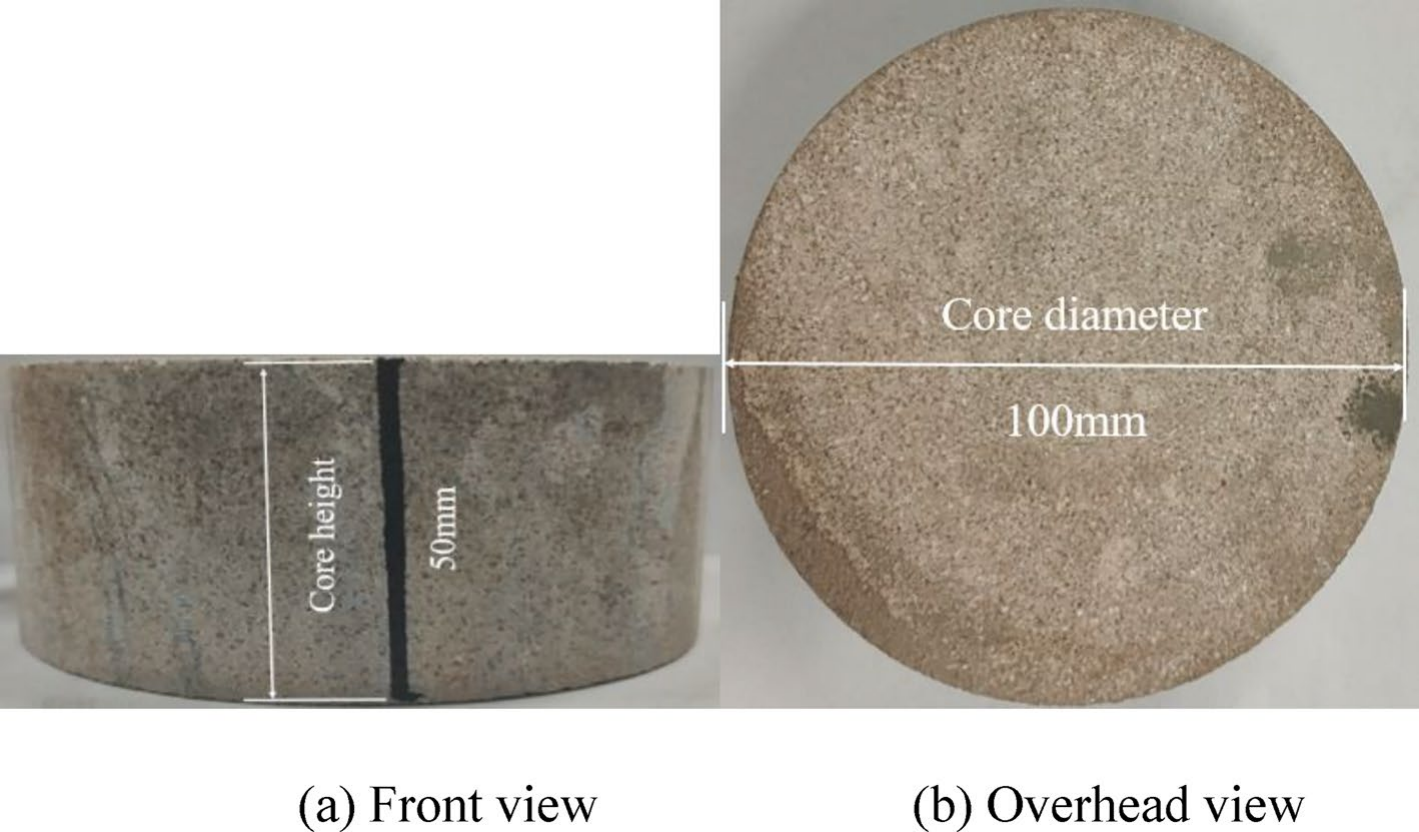
Image of the simulated rock core.

In this study, a simulated cylindrical core of a weakly cemented formation was prepared using quartz sand (250–300 mesh), clay, and water in a ratio of 4:1:0.8. Then, the same pressure (12 MPa) was used to press and prepare the core sample, which was then cured at room temperature for 24 h. Finally, drying treatment was performed to ensure consistent core water saturation, cylindrical core as shown in Fig. 1.

**Figure 2:**
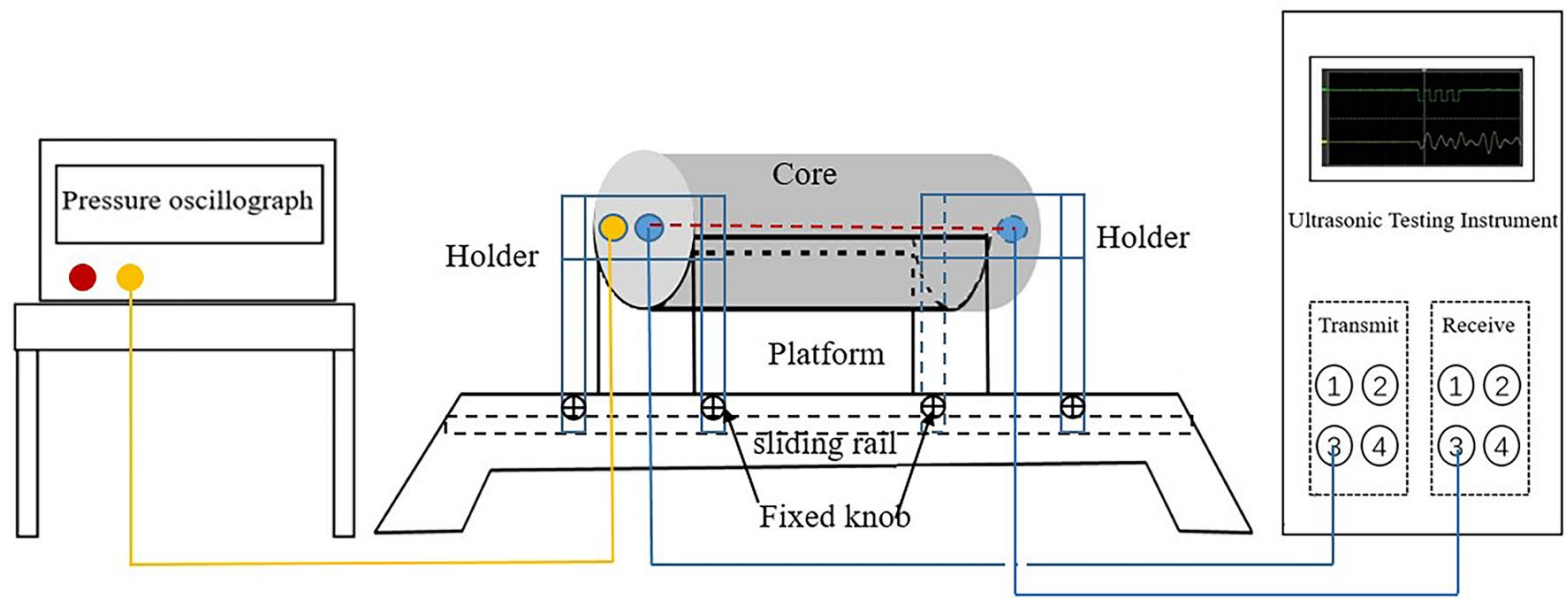
Ultrasonic acoustic response experimental platform.

After the rock core was prepared, ultrasonic testing was carried out. As shown in Fig. 2, an ultrasonic acoustic response experimental platform was used for experimental response testing, mainly consisting of instruments such as ultrasonic testing instruments, a pressure oscilloscope, and holding platforms. It is worth mentioning that the ultrasonic testing instrument (HTY-M, purchased) could use multiple sound wave frequencies (60 kHz, 400 kHz, 500 kHz, 750 kHz and 1000 kHz). The holding platform was independently designed and built, including U-shaped platform, holder and fixed knob. The core was clamped in place using a U-shaped platform, and a sliding rail and fixed knob were added to clamp the core with the holder. The pressure oscilloscope (FNIRSI-1014D, purchased) was used to ensure that the pressure between the test chamber clamp and the rock core was consistent, reducing experimental errors.

The test steps were as follows:

1. The core was cut and polished into two parts of the same height, and the height L of the core was measured. One part was used to measure the physical parameters, and the end surface of the other part was ground until it was smooth. Vaseline was then smeared on the sample to be measured.
2. One end of the transducer was connected to the same receiving and transmitting groups of the ultrasonic tester, while the other end was connected to the probe surface after smearing it with Vaseline. The inherent ultrasonic wave time T_1_ of the probe was tested.
3. The sample was placed on a core holder and clamped. The squeezing force between the core and transducer was read using the pressure oscilloscope to maintain a constant squeezing force for each test.
4. The ultrasonic curve was obtained using an ultrasonic tester, the time T_2_ of the first wave was read, and the velocity of the first wave was calculated using the Formula v = L/(T_2_ − T_1_).

## Results and discussion

The propagation of waves in layered media can be described using ray theory or equivalent medium theory^11^. This description considers the ratio of the wavelength to the dielectric layer thickness as a control parameter. The measurement speed is related not only to the composition of the target medium to be reflected but also to the measurement scale and geometric scale of the target geological body.

When measured over a certain frequency range, the P-wave velocity of the rock changes with a change in frequency, commonly known as dispersion^11^. The relation between velocity and frequency can be expressed by the frequency dispersion, which depicts the degree of change in the P-wave velocity under different frequency conditions. This parameter measures the relative difference between velocities in the same medium caused by the change in the measurement frequency and the velocity value before the change.

### Effect of the ultrasonic frequency on the P-wave velocity

**Figure 3:**
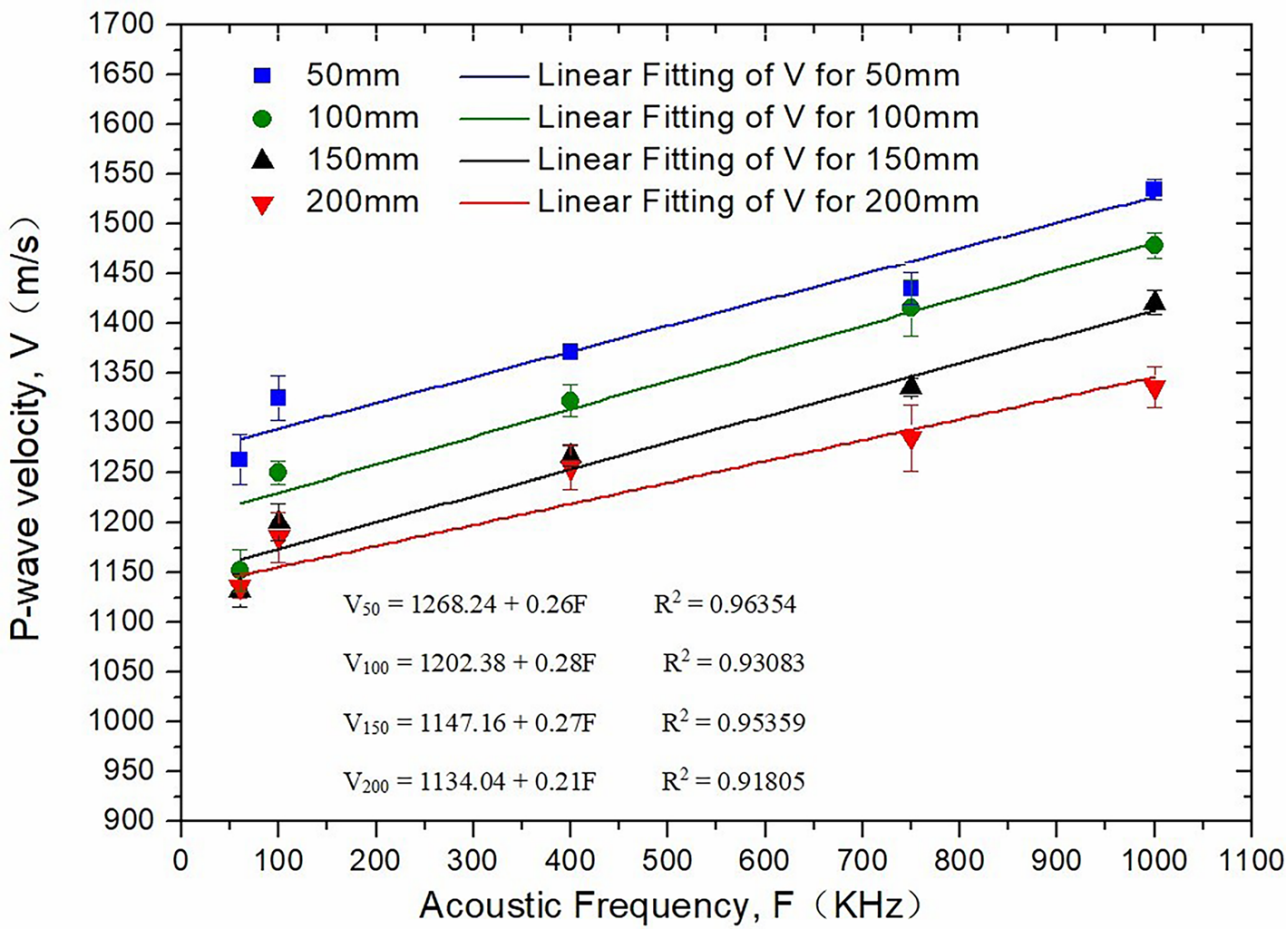
Effect of ultrasonic frequency on P-wave velocity.

Figure 3 presents the effect of the ultrasonic frequency on the P-wave velocity. For the same core height (50 mm), the P-wave velocity increases with increasing ultrasonic frequency. In the process of ultrasonic propagation, attenuation and dispersion are two different processes that occur simultaneously^12,13^. The main cause of ultrasonic attenuation is the presence of a pore fluid. The relative motion between the fluid and solid leads to the dissipation of ultrasonic energy and attenuation of fluid relative to the skeletal flow and fluid viscosity. Furthermore, ultrasonic dispersion of the rock results from the combined effect of scattering and viscous absorption. In the dry state, ultrasonic dispersion in the core was mainly affected by the scattering effect, and the frequency therefore decreased. The dispersion was more evident when the scattering effect was significant. The smaller the ultrasonic frequency was, the more severe the dispersion and the smaller the P-wave velocity.

### Effect of particle size (porosity) on P-wave velocity

The particle size directly affects porosity and indirectly affects P-wave velocity^14,15^. Hence, in this paper, 100 mm high cores were prepared using quartz sand with 5 different particle sizes (48–75 μm, 75–106 μm, 106–150 μm, 150–180 μm and 180–270 μm) and metakaolin in the same proportion (core height: 50 mm). The porosities of cores prepared with different particle sizes are shown in Table 1.

**Table 1:** Porosity under different particle sizes.

Case	Particle size, s (μm)	Porosity, P (%)
1	48–75	14.95
2	75–106	15.60
3	106–150	16.20
4	150–180	18.55
5	180–270	19.06

**Figure 4:**
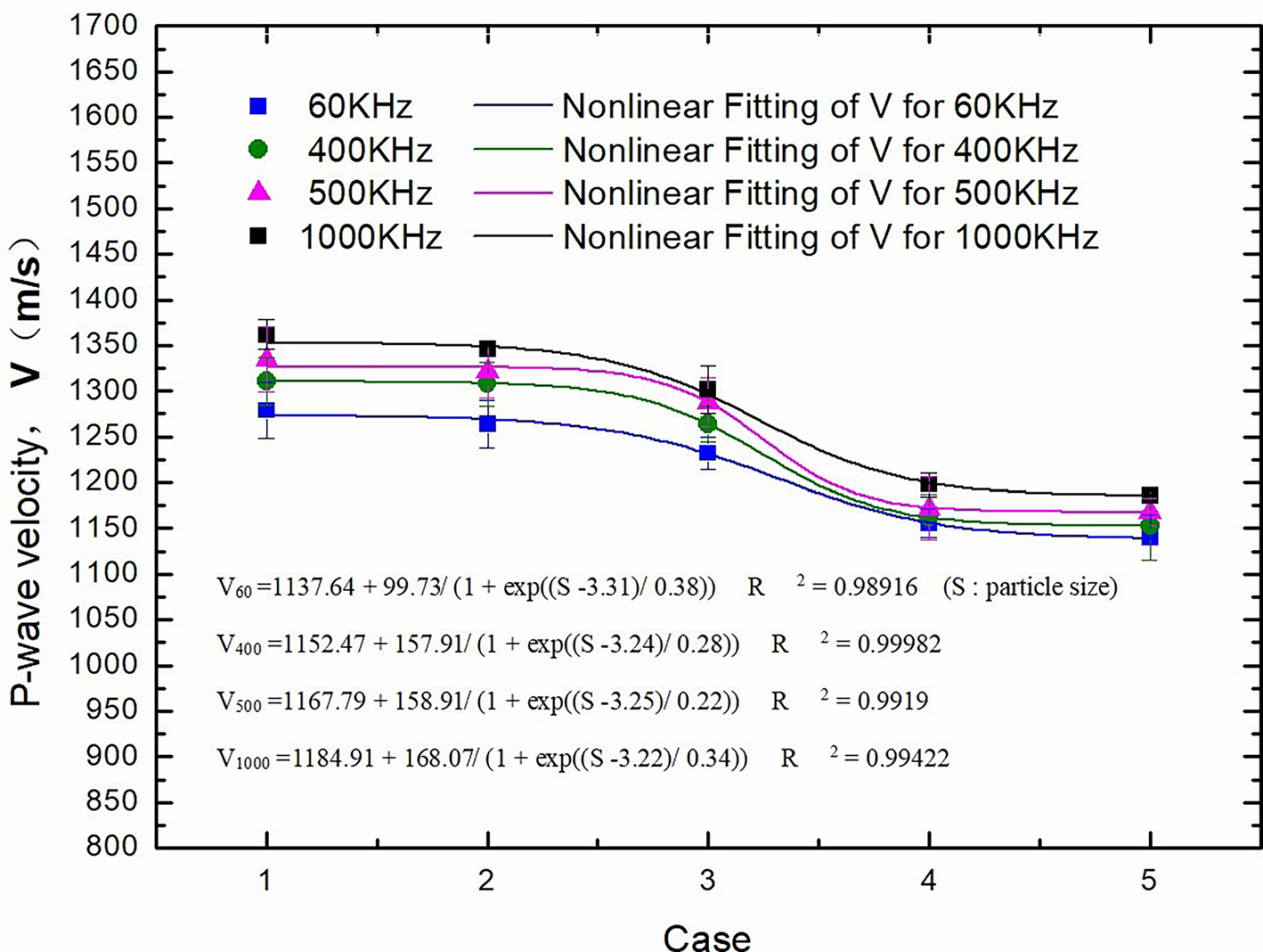
Effect of particle size (porosity) on P-wave velocity.

The effect of different core particle sizes on the P-wave velocity is shown in Fig. 4. In general, a larger particle size corresponds to a smaller P-wave velocity. This is observed because under the same compaction pressure during the production of the simulation core, a larger particle size implies larger pores between particles. In the process of penetrating the core, ultrasonic waves need to cross different media, resulting in greater energy loss. Another important observation is that when the particle size is within the ranges of 48–106 μm and 150–270 μm, the change in P-wave velocity is small, whereas when the particle size is within the range of 106–150 μm, the change in P-wave velocity is large. The reason behind this is that when the particle size is small, the packing between particles is compact, and the compaction pressure has little impact on the pore space. Similarly, when the particle size is large, the particles are loosely packed, and the compaction pressure has little impact on the pore space. However, when the particle size is within the range of 106–150 μm, the compaction pressure can effectively reduce the pore space between particles, leading to significant fluctuations in P-wave velocity measurements.

### Effect of density on P-wave velocity

The density of rock reflects its various properties^16^, including mineral composition and cementation type. Ultrasonic waves have different propagation speeds in different solid media, particularly in the presence of different mineral components. In this study, core samples with different densities were prepared by adding glass beads based on the original core formula, as shown in Table 2. The glass beads are only used for experiments in this chapter to adjust core density.

**Table 2:** Formulas with different proportions of glass beads.

Quartz sand (g)	Metakaolin (g)	Water (ml)	Glass beads (g)	Density (g/cm^3^)
			0	2.42
			50	2.32
2000	500	400	100	2.24
			150	2.15
			200	2.06

**Figure 5:**
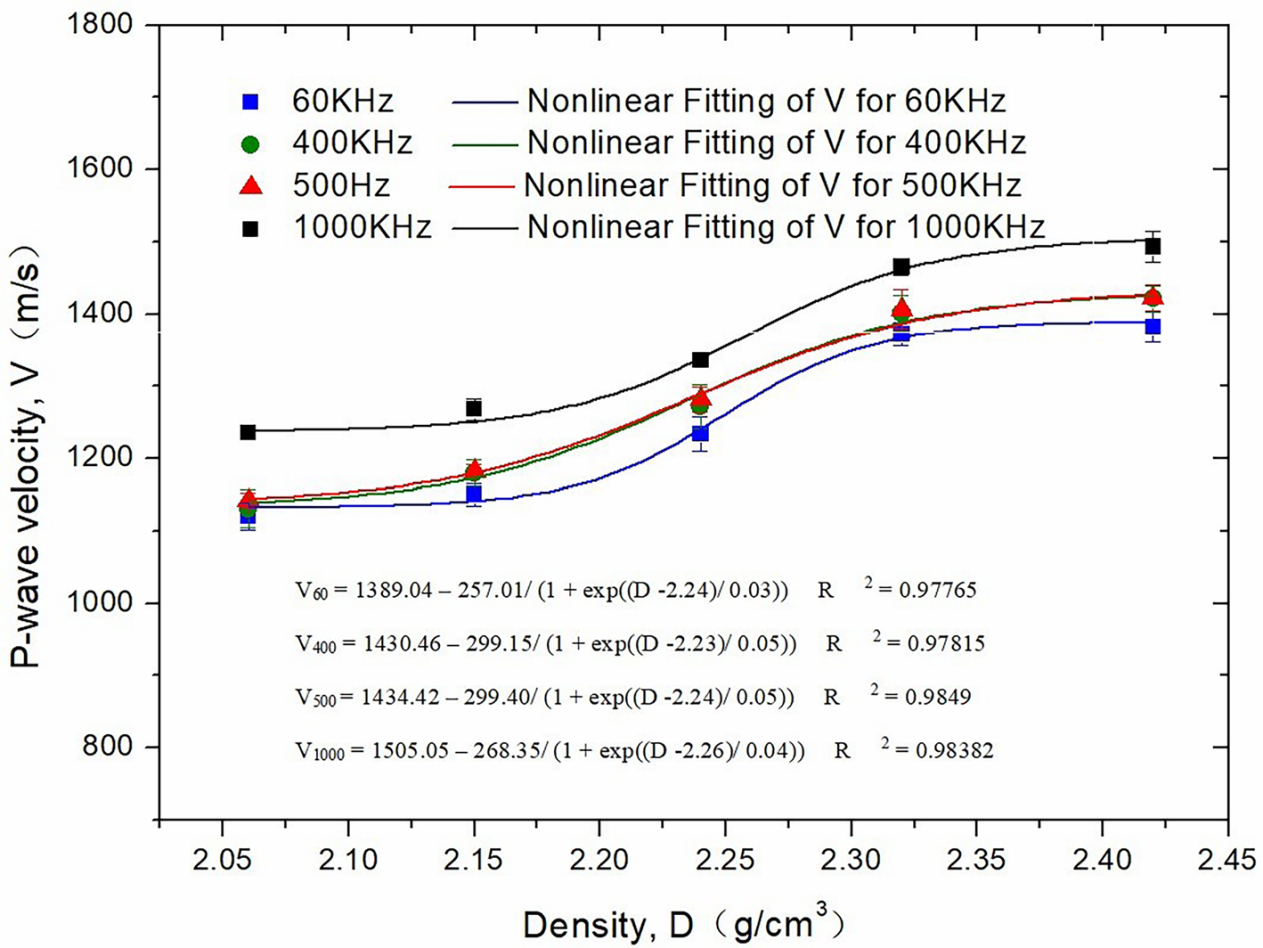
Effect of density on P-wave velocity.

Figure 5 shows the P-wave velocity of 100 mm high core samples at different densities, which indicates a nonlinear multisegment relationship with the increase in density. When the density is within the range of 2.06–2.15 g/cm^3^, the P-wave velocity rises slowly with increasing density. However, as the density enters the range of 2.15–2.32 g/cm^3^, the P-wave velocity increases significantly and tends to become stable gradually as the density enters the range of 2.32–2.42 g/cm^3^. This can be explained as follows: The propagation of ultrasonic waves depends on the medium, and a greater density of the medium particles provides faster transfer of the ultrasonic energy, which is confirmed by the higher speed of the ultrasonic wave. At the same time, within a certain density range, the P-wave velocity increases exponentially with an increase in density; beyond this range, the effect of the increase in density on the P-wave velocity weakens gradually.

### Effect of compressive strength on P-wave velocity

The geological strength of weakly cemented formations also affects the P-wave velocity^17^. To analyse the effect of compressive strength on P-wave velocity, different contents (0 g, 50 g, 100 g, 150 g and 200 g) of cement were added to the core formula to prepare simulated cores with different strengths, and the uniaxial compressive strengths corresponding to different simulated rock cores are shown in Table 3. The glass beads are only used for experiments in this chapter to adjust core density. The cement is only used for experiments in this chapter to adjust compressive strengths.

**Table 3:** Uniaxial compressive strengths of rock cores with different cement contents.

Case	Quartz sand (g)	Metakaolin (g)	Water (ml)	Cement (g)	Compressive strength (MPa)
1				0	4.12
2				50	4.53
3	2000	500	400	100	4.75
4				150	5.25
5				200	5.68

**Figure 6:**
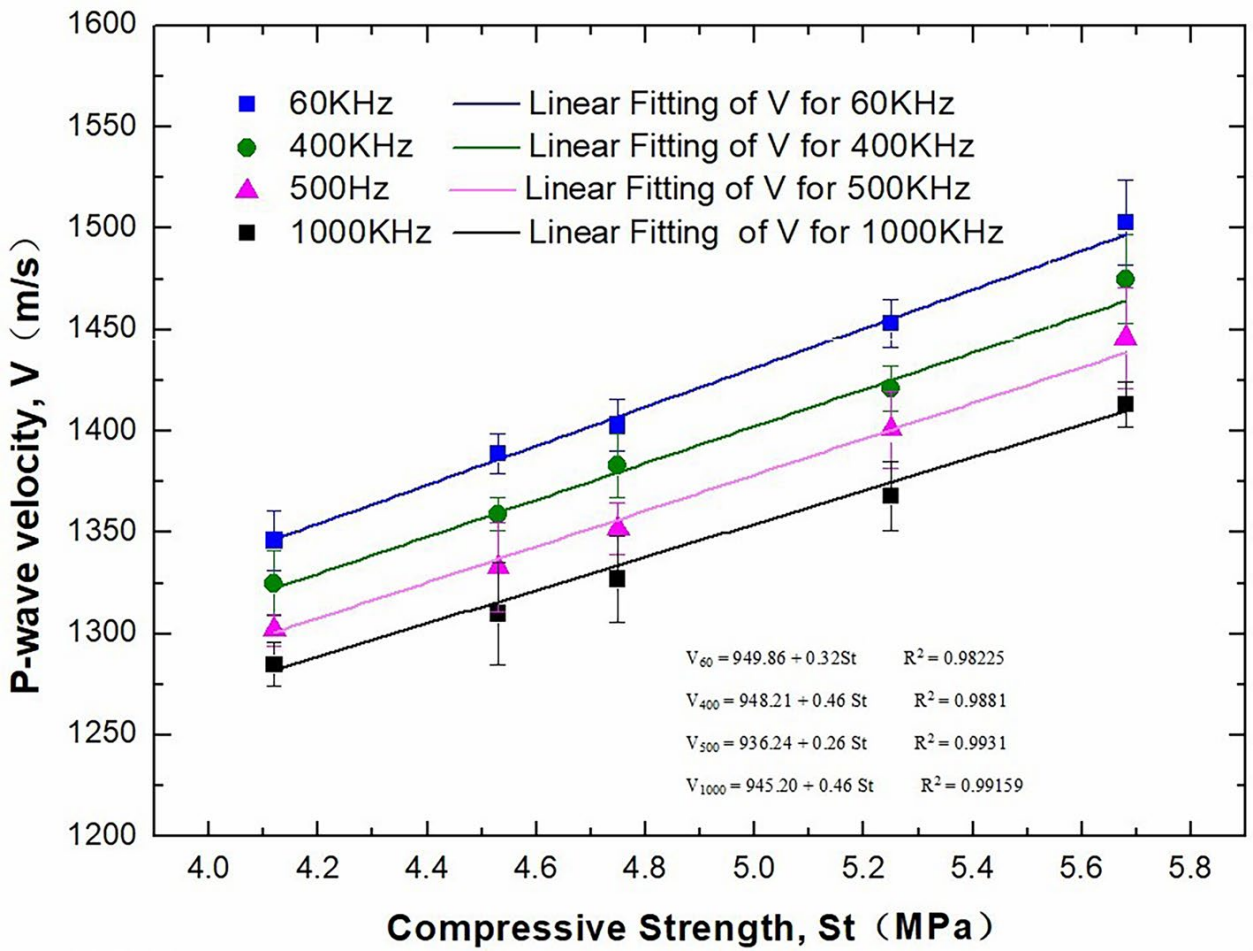
Effect of compressive strength on P-wave velocity (500 kHz).

Figure 6 shows the P-wave velocities of 25 mm high core samples at different compressive strengths, and the ultrasonic frequency used for testing is 500 kHz. The higher the compressive strength of the core samples is, the greater the P-wave velocity. The greater the compressive strength is, the greater the elastic modulus of the core sample; this means the compactness of the ultrasonic transmission medium is greater, so the measured P-wave velocity is greater.

### Ultimate height of the core measured at different ultrasonic frequencies

The P-wave velocity is related not only to geological properties but also to geological scale^18^. To reveal the effect of geological scale on P-wave velocity, the simulated rock core was cut into different heights (25 mm, 50 mm, 100 mm, 150 mm and 200 mm), as shown in Fig. 1.

**Figure 7:**
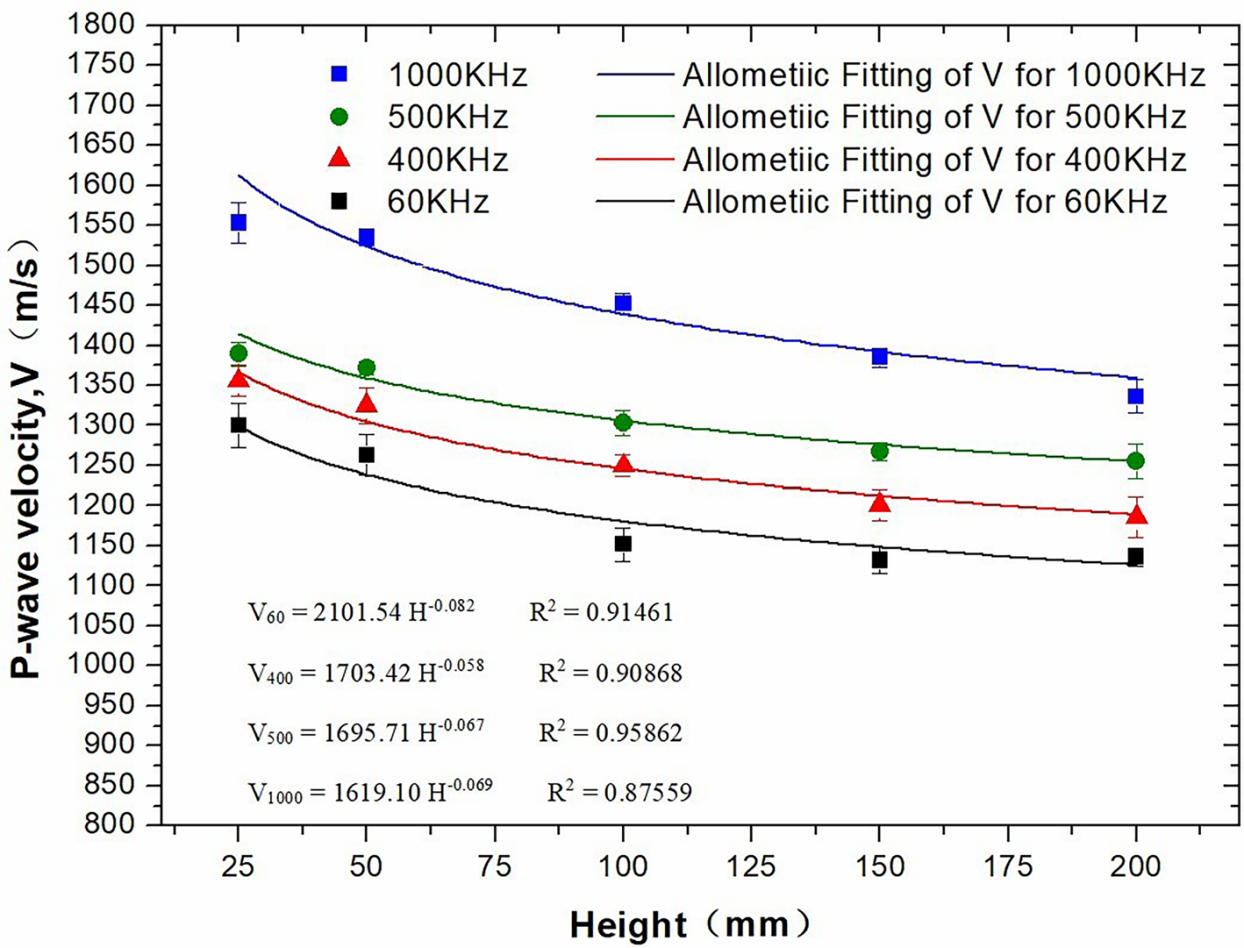
Effect of core height on P-wave velocity.

Figure 7 shows the effect of different core heights (distance between the two circular end faces of a cylindrical core) on the P-wave velocity. It can be seen from the figure that under the same ultrasonic frequency, an increase in the core height results in an initial decrease in the P-wave velocity before stabilizing. The reason for this change is that with an increase in the core length, the sound waves with rich frequency components emitted by the transducer gradually lose their high-frequency components. Thus, the frequency of the received wave continuously decreases at first, but the received waveform then gradually becomes smooth and gentle, and as the first arrival point of the sound wave approaches, the measured sound time increases and results in a decrease in the calculated sound speed. The results indicate that within a certain testing length range, the P-wave velocity decreases with increasing testing length and gradually approaches a fixed value.

In addition, it can be seen in Fig. 7 that a bigger core height at higher frequencies corresponds to stronger attenuation of the P-wave velocity, whereas a smaller core height at lower frequencies corresponds to weaker attenuation of the P-wave velocity.

**Figure 8:**
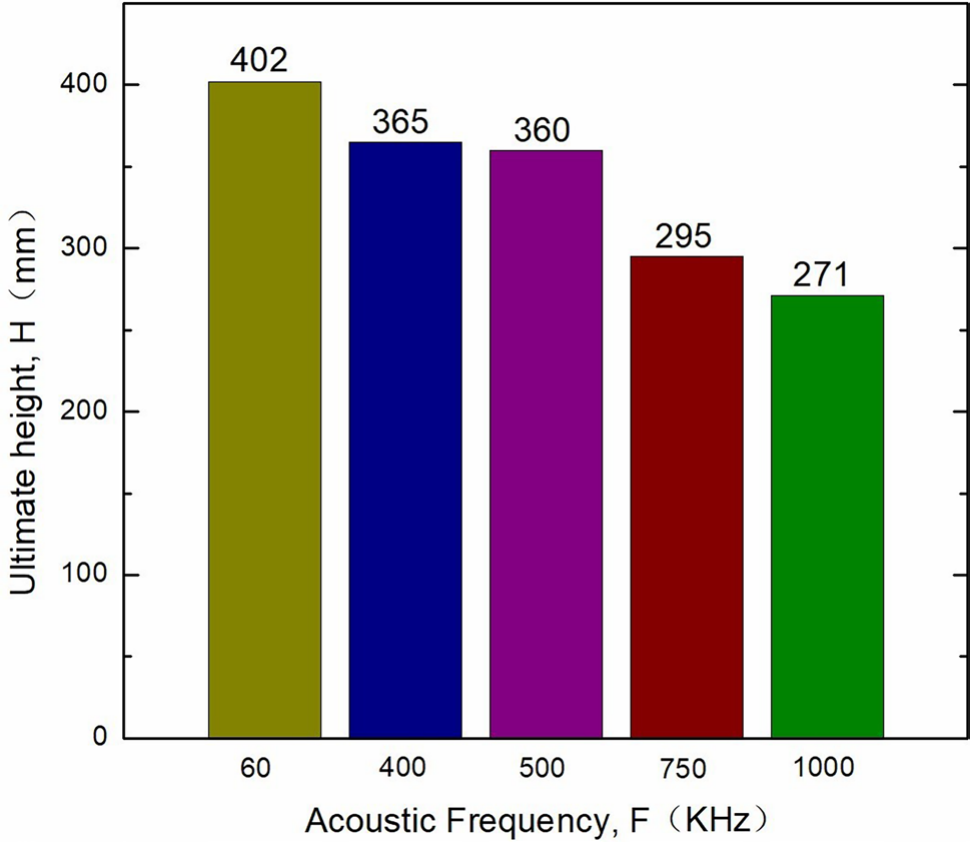
Ultimate height of the core measured at different ultrasonic frequencies.

In view of the above observations, experiments were conducted on the ultimate height of ultrasonic penetration of rock cores at different frequencies. This research adopts the method of cutting during ultrasonic testing; that is, using the same core, 10 mm is removed at the end face of the same core each time, and ultrasonic testing is conducted at different frequencies until the ultimate height of the core corresponding to different frequencies is determined. Figure 8 shows the ultimate height of the rock core, which can be measured at different ultrasonic frequencies. The ultimate height of the core that can be tested at a frequency of 1000 kHz is 271 mm, and ultrasonic testing cannot be carried out if the ultimate height is exceeded. However, at a frequency of 60 kHz, the ultimate height of the core that can be tested reaches 402 mm. This shows that ultrasonic energy attenuation is significant when the ultrasonic frequency is high.

## Enlightenment and extension research

**Figure 9:**
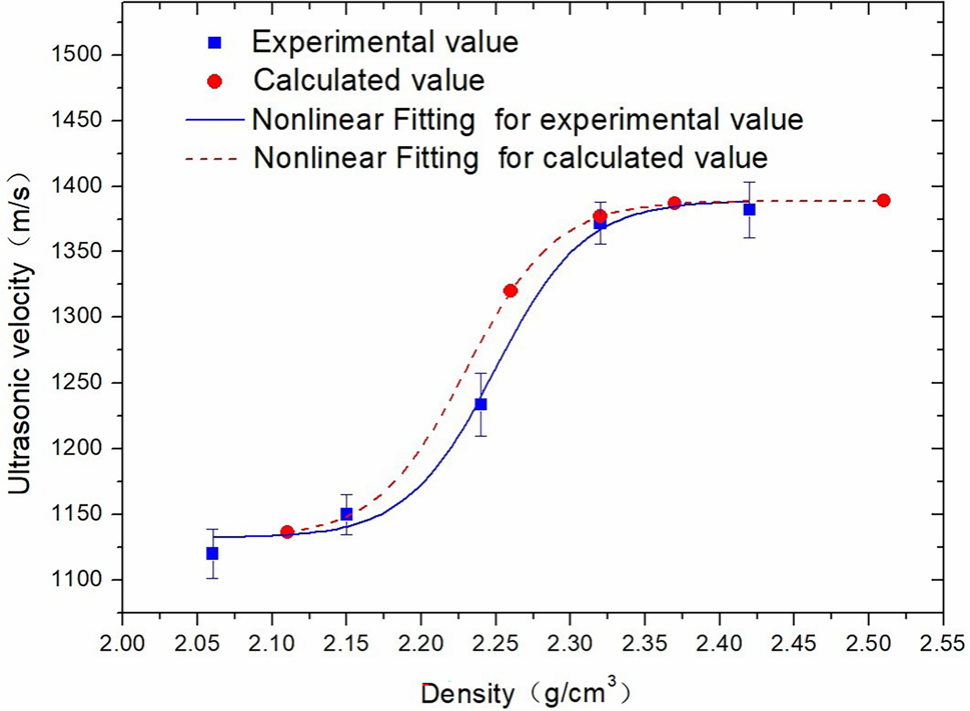
Comparison of experimental data and calculated data for density values.

In this study, the P-wave velocity of the same core was tested experimentally and calculated theoretically, and a comparative analysis was performed, as shown in Fig. 9. An ultrasonic frequency of 60 kHz and a core height of 500 mm were used for the experimental tests. The theoretical calculation formula adopted the empirical data fitting relations of Formula (1) under a 60 kHz ultrasonic frequency, as discussed in section “Effect of density on P-wave velocity”:

1 $$ {\text{V}} = 1389.04  - 257.01/(1  + \exp (({\text{D}} - 2.24)/0.03))\quad {\text{R}}^{2} =  0.97765 $$

where *V* is the P-wave velocity, m/s, and D is the density, g/cm^3^. The measured P-wave velocity was then substituted into the formula to calculate the density value. The results indicate that the experimental data curve has the same trend as the theoretical calculation curve, and the difference between the two is less than 10%. Therefore, it is feasible to establish a correlation between ultrasonic response and formation physical properties. In subsequent logging operations, after measuring the ultrasonic velocity, this relationship can be used to indirectly obtain the physical properties of the formation.

It is worth mentioning that this method still has limitations, this study was conducted in the laboratory, with homogeneous experimental materials and stable experimental environment, and there were errors for complex actual formations, but it is still a new approach.

## Conclusions

In this study, experiments to examine the influence of different factors on P-wave velocity were conducted for weak gel formation. The following conclusions were drawn.

1. The P-wave velocity is related to the frequency of the ultrasound itself. The P-wave velocity varies linearly with ultrasonic frequency, and with an increase in ultrasonic frequency, the P-wave velocity increases.
2. The P-wave velocity is related to geological properties.
  The P-wave velocity exhibits a nonlinear relationship with particle size (porosity) and density. The P-wave velocity decreases with increasing particle size (porosity) and increases with increasing density. The P-wave velocity exhibits a nonlinear relationship with compressive strength, and it increases with increasing compressive strength.
3. The P-wave velocity is related to the measured geological scale. As the sample length (core height) increases, it first decreases and then gradually approaches a fixed value. In addition, each frequency corresponds to the ultimate height of one core.
4. Due to the high difficulty of obtaining on-site materials and the difficulty of on-site construction, the experimental results in this article are all based on indoor simulation experiments, which have certain limitations. The use of ultrasonic response to correlate with formation properties to obtain material data also has limitations, but it is still a method of ultrasonic detection of weakly cemented formations in deep water.

## Data Availability

The datasets generated during and/or analyzed during the current study are available from the corresponding author on reasonable request.
